# Why is primary eye health care needed?

**Published:** 2022-03-01

**Authors:** Clare Gilbert, Mapa Prabhath Piyasena

**Affiliations:** 1 Professor of International Eye Health: International Centre for Eye Health, London School of Hygiene and Tropical Medicine, London, UK.; 2 Postdoctoral Research Fellow in Global Eye Health: Centre for Public Health, Queen's University Belfast, Belfast, Northern Ireland.

**Eye conditions are common in the community; primary eye care can address many of them.**

When thinking about delivering eye care services at the primary care level, it is important to consider the eye care needs of the population. Adults and children with eye conditions can be divided into four separate groups (Table 1):^1^

1. People with visual impairment, for whom treatment will improve or restore vision
2. People with visual impairment, whose visual impairment is not reversible
3. People without visual impairment, who need treatment to prevent visual impairment and/or death
4. People without visual impairment, who are not at risk of becoming visually impaired but have symptoms that must be treated.

It is important to remember that most people do not have an eye condition, nor are they visually impaired. Health promotion and specific preventive measures are needed to maintain their eye health and good vision.

**Table 1:** Examples of eye conditions in each group

**Group**	**Eye conditions**	**Interventions needed**
**Already visually impaired**		
**Group 1** Treatment can improve or restore vision	Uncorrected refractive errors, cataract and presbyopia	Cataract surgery and optical correction
**Group 2** The vision impairment is not reversible	End-stage glaucoma, diabetic retinopathy, and retinopathy of prematurity; congenital anomalies; dense corneal scarring; optic atrophy	Vision rehabilitation
**Not visually impaired**		
**Group 3** Treatment is needed to prevent vision impairment (or death)	Early/undetected glaucoma, diabetic retinopathy, age-related macular degeneration (wet form),^*^ retinoblastoma, and conjunctival cancers	Early detection and management with life-long care
**Group 4** Vision impairment is highly unlikely, but symptoms must be treated	Conjunctivitis, dry eye, lid infections	Treatment as appropriate, often topical

* Wet AMD is the only type of age-related macular degeneration which can currently be treated.

## How many people have these different eye conditions?

Estimating the number of people of all ages who fall into these different groups is challenging, as surveys do not always collect relevant data. More is known about Group 1 and 2 conditions, and some estimates can be made for Group 3 and 4 conditions.

It is useful to estimate the number of people affected in a total population of 100,000 people, as this is the size of the population served by one or more health centres or polyclinics in most low- and/or middle-income countries.

The numbers given in Table 2 are based on the following assumptions.

1. ‘Uncorrected presbyopia’ is an estimate of the number per 100,000 population in each region who need correction for presbyopia (near vision impairment). An estimated 80% of the global population aged 40 years and above have presbyopia.
2. ‘Undetected glaucoma’ is an estimate of the number of people with undiagnosed glaucoma in each region, minus those who are already blind or visually impaired due to glaucoma (they are included in Group 2). An estimated 3% of the global population aged 40 years and above have glaucoma.
3. ‘At risk of diabetic retinopathy’ is based on the International Diabetes Federation's estimates of the number of people with diabetes aged 20 years and above (all of whom are at risk of diabetic retinopathy), minus those who are already blind or visually impaired due to diabetic retinopathy (who are included in Group 2).
4. 5% is a minimum estimate of the prevalence of all non-visually impairing conditions such as conjunctivitis, dry eyes, lid infections, etc. This figure could be as high as 10%, but more data are needed.

**Figure:**
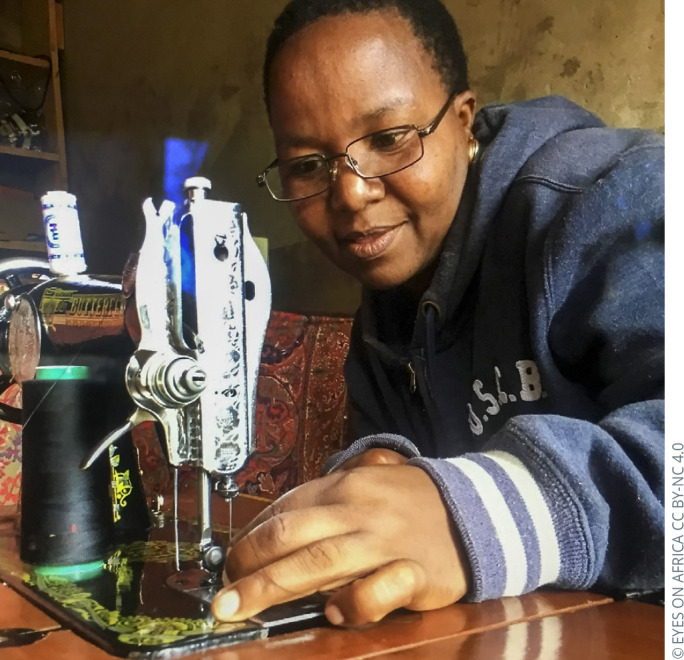
Refractive errors are responsible for most of the need for eye care services at primary level.

**Table 2:** Estimates of the number of people in a population of 100,000 with an eye condition in each of the four groups (by region).

	**Latin America (per 100,000)**	**Asia (per 100,000)**	**Africa (per 100,000)**
**Group 1 conditions**	**9,500**	**19,000**	**13,500**
Cataract^*^	1,000	1,500	500
Uncorrected refractive error (distance)^*^	2,500	2,500	1000
Uncorrected presbyopia^**^^1^	6,000	15,000	12,000
**Group 2 conditions**	**1,200**	**1,000**	**800**
Blind/VI from glaucoma, AMD, DR, other conditions^*^	1,200	1,000	800
**Group 3 conditions**	**1,200**	**1,500**	**700**
Undetected glaucoma^2^	750	800	500
At risk of diabetic retinopathy^3^	450	700	200
**Group 4 conditions**	**5,000**	**5,000**	**5,000**
Non-visually impairing eye condition (5% of the population)^4^	5,000	5,000	5,000
**Total (%) affected in a population of 100,000**	**16,900 (17%)**	**26,500 (27%)**	**20,000 (20%)**

* Presenting visual acuity of less than 6/18 in the better-seeing eye.^1^

** Presenting near acuity of < N61 (ICD-11 definition).^2^

### What do these numbers mean?

The estimates in Table 2 suggest that more than 1 in 4 people living in communities in Asia (27%) have an eye care need, compared to 1 in 5 in Africa (20%) and 1 in 6 in Latin America (17%). The differences between regions reflect:

1. Differences in the age structure of the population
2. Variation in the prevalence of the conditions
3. The extent to which people with these eye conditions have already accessed services.

Refractive errors (both distance and near) make up the majority of the conditions listed. However, the numbers given for refractive errors do not include individuals with a presenting acuity of <6/12 to 6/18 in the better eye, as data on the causes of visual impairment in this category are not well known. However, we can be reasonably certain that uncorrected refractive errors are the main cause. It is also important to note that the numbers do not include those who already have spectacles for distance and/or near vision, who will need ongoing services.

## What role can primary eye health care play in addressing these conditions?

### Group 1 conditions

#### Uncorrected refractive errors, cataract, and presbyopia

Primary health care workers can identify people with distance vision impairment by measuring presenting distance visual acuity. Examining the eye with a torch will enable them to differentiate cataract from other causes, and testing visual acuity with a pinhole will detect those with uncorrected refractive errors. These two procedures alone will identify between 65% (in Africa) and 80% (in Asia) of the people with vision impairment in their catchment population.

Measuring presenting near visual acuity with both eyes open will detect presbyopia. In some settings, primary health care workers are trained and able to dispense presbyopic correction as long as distance visual acuity is normal; if not, referral will be needed.

### Group 2 conditions

#### End-stage glaucoma, diabetic retinopathy, and retinopathy of prematurity; congenital anomalies; dense corneal scarring; optic atrophy

People who need to be referred to an eye care professional for assessment prior to vision rehabilitation are those in whom:

- The visual acuity is less than 6/18 in the better eye
- The vision does not improve to better than 6/18 in either eye with a pinhole
- Cataract has been excluded.

Vision rehabilitation can help to improve the quality of life of the people affected and help them to maintain independence.

### Group 3 conditions

#### Early or undetected glaucoma, diabetic retinopathy, age-related macular degeneration (wet form),* retinoblastoma, and conjunctival cancers

Detecting glaucoma and diabetic retinopathy at the primary level is far more challenging than Group 1 conditions, as the diagnosis requires more sophisticated equipment and clinical skills. However, primary health care workers can play an important role, by asking adults whether they have diabetes, or whether a family member has glaucoma. If so, they should be referred for examination.

### Group 4 conditions

#### Conjunctivitis, dry eye, and lid infections

Primary eye care workers can play an important role in detecting and managing less complex eye conditions which can cause troublesome symptoms, such as conjunctivitis and dry eye. To do this, they will require skills in detecting the condition, knowledge on how to treat it, and access to relevant medication. Follow-up will also be needed to ensure the condition is getting better. If not, referral will be required.

## Maintaining good vision and healthy eyes

**Figure 1:**
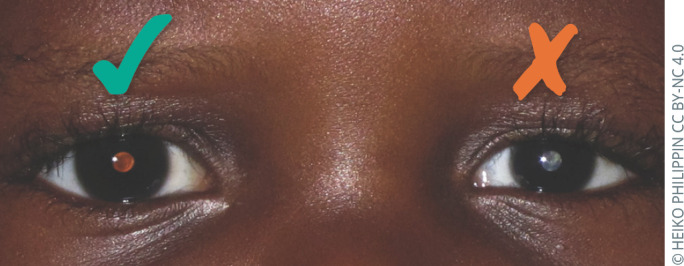
Right eye: the normal red reflex. Left eye: the wrong colour in a red reflex could indicate a serious condition. The child in this image has a cataract in the left eye. Refer the child to a specialist.

In all regions, infants require measles immunisation at 9 months of age. In some regions, child health policies include vitamin A supplementation for preschool age children and ocular prophylaxis at birth to prevent conjunctivitis of the newborn. Primary health care workers can also carry out red reflex testing of newborns within 6–8 weeks of birth and at older ages to detect cataract and retinoblastoma (Figure 1).^3^ All infants who fail the red reflex test should be referred urgently.
